# Impact of cluster headache on employment status and job burden: a prospective cross-sectional multicenter study

**DOI:** 10.1186/s10194-018-0911-x

**Published:** 2018-09-03

**Authors:** Yun-Ju Choi, Byung-Kun Kim, Pil-Wook Chung, Mi Ji Lee, Jung-Wook Park, Min Kyung Chu, Jin-Young Ahn, Byung-Su Kim, Tae-Jin Song, Jong-Hee Sohn, Kyungmi Oh, Kwang-Soo Lee, Soo-Kyoung Kim, Kwang-Yeol Park, Jae Myun Chung, Heui-Soo Moon, Chin-Sang Chung, Soo-Jin Cho

**Affiliations:** 1 Department of Neurology, Presbyterian Medical Center, Jeonju, South Korea; 2 Department of Neurology, Eulji Hospital, Seoul, South Korea; 3 Department of Neurology, Kangbuk Samsung Hospital, Sungkyunkwan University School of Medicine, Seoul, South Korea; 4 Department of Neurology, Samsung Medical Center, Seoul, South Korea; 5 Department of Neurology, Uijeongbu St.Mary’s Hospital, Uijeongbu, South Korea; 6 Department of Neurology, Severance Hospital, Seoul, South Korea; 7 Department of Neurology, Seoul Medical Center, Seoul, South Korea; 8 Department of Neurology, Bundang Jesaeng General Hospital, Daejin Medical Center, Seongnam, South Korea; 9 Department of Neurology, Ewha Womans University of Medicine, Seoul, South Korea; 10 Department of Neurology, Chuncheon Sacred Heart Hospital, Chuncheon, South Korea; 11 Department of Neurology, Korea University College of Medicine, Seoul, South Korea; 12 Department of Neurology, Seoul St. Mary’s Hospital, Catholic University of Korea College of Medicine, Seoul, South Korea; 13 Department of Neurology and Institute of Health Science, Gyeongsang National University College of Medicine, Jinju, South Korea; 14 Department of Neurology, Chung-Ang University Hospital, Seoul, South Korea; 15 Department of Neurology, Inje University College of Medicine, Seoul, South Korea; 16 Department of Neurology, Dongtan Sacred Heart Hospital, Hallym University College of Medicine, Keun Jae Bong-gil 7, Hwaseong, Gyeonggi-do 18450 South Korea

**Keywords:** Cluster headache, Disability, Employment, Occupation, Sick leave, Work

## Abstract

**Background:**

Cluster headaches (CH) are recurrent severe headaches, which impose a major burden on the life of patients. We investigated the impact of CH on employment status and job burden.

**Methods:**

The study was a sub-study of the Korean Cluster Headache Registry. Patients with CH were enrolled from September 2016 to February 2018 from 15 headache clinics in Korea. We also enrolled a headache control group with age-sex matched patients with migraine or tension-type headache. Moreover, a control group including individuals without headache complaints was recruited. All participants responded to a questionnaire that included questions on employment status, type of occupation, working time, sick leave, reductions in productivity, and satisfaction with current occupation. The questionnaire was administered to participants who were currently employed or had previous occupational experience.

**Results:**

We recruited 143 patients with CH, 38 patients with other types of headache (migraine or tension-type headache), and 52 headache-free controls. The proportion of employees was lower in the CH group compared with the headache and headache-free control groups (CH: 67.6% vs. headache controls: 84.2% vs. headache-free controls: 96.2%; *p* = 0.001). The CH group more frequently experienced difficulties at work and required sick leave than the other groups (CH: 84.8% vs. headache controls: 63.9% vs. headache-free controls: 36.5%; *p* <  0.001; CH: 39.4% vs. headache controls: 13.9% vs. headache-free controls: 3.4%; *p* <  0.001). Among the patients with CH, sick leave was associated with younger age at CH onset (25.8 years vs. 30.6 years, *p* = 0.014), severity of pain rated on a visual analogue scale (9.3 vs. 8.8, *p* = 0.008), and diurnal periodicity during the daytime (*p* = 0.003). There were no significant differences with respect to the sick leave based on sex, age, CH subtypes, and CH recurrence.

**Conclusions:**

CH might be associated with employment status. Most patients with CH experienced substantial burdens at work.

**Electronic supplementary material:**

The online version of this article (10.1186/s10194-018-0911-x) contains supplementary material, which is available to authorized users.

## Background

Cluster headache (CH) refers to trigeminal autonomic cephalalgia characterized by recurrent, severe unilateral pain and ipsilateral autonomic symptoms and has a negative impact on patient life [1]. Previous studies have shown that patients with CH report restrictions in daily living, difficulties in social-activity participation, family life, and housework; and overall life changes [2, 3]. The incidence of CH is high among young men; therefore, CH may have a significant impact on employment. A previous study showed that 30% of patients experienced absenteeism due to CH [3]. Rozen et al. reported that approximately 20% of patients with CH experienced job loss and that 8% were unemployed or were receiving disability payments [4]. However, information regarding associations among occupational status, reductions in job productivity, and sick leave with the characteristics of CH is currently limited.

In a large population-based study with patients who had headaches, 31% of participants reported that their work level was reduced by > 50% due to headaches during working hours. In addition, the mean number of absent days due to headaches was 4.2 in the past year. More than half of the patients who experienced these difficulties in the workplace reported that they were due to migraines [5]. In a Spanish study, individuals with migraines showed the lowest productivity and highest loss of workday equivalents [6]. Migraine headaches can cause serious problems; however, CH is also severe and can be expected to cause many work-related difficulties.

In this study, we analyzed the effect of CH on employment status, type of occupation, working time, difficulties including sick leave and decreases in productivity, and satisfaction with current employment. We compared patients with CH to patients with migraine or tension-type headaches (TTH) and a headache-free control group. In addition, we investigated anxiety, depression, and stress levels and analyzed all these factors as predictors of difficulties at work and sick leave.

## Methods

### Study design and patients

**Fig. 1:**
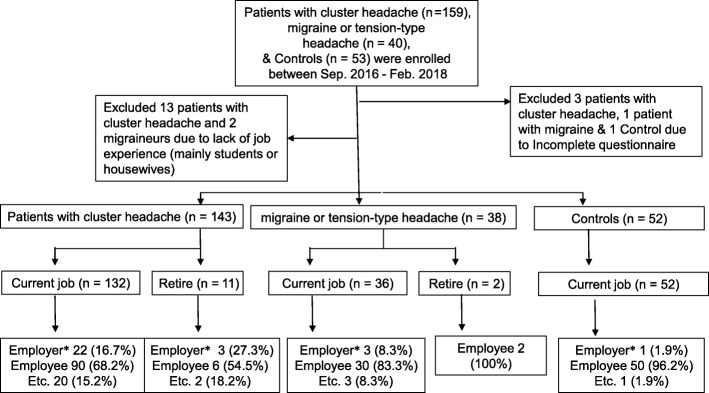
Flow chart depicting the participation of subjects. *Employer included self-employment

The Korean Cluster Headache Registry Study is a prospective, cross-sectional, multicenter registry study that enrolled consecutive patients with CH from 15 hospitals (13 university hospitals: eight tertiary and five secondary referral hospitals and two secondary referral general hospitals) in Korea. This study used data from patients enrolled between September 2016 and February 2018. Inclusion criteria were: CH diagnosis, episodic, chronic, or probable CH; adult age (≥ 19 years), and full understanding and agreement of the study protocol. A diagnosis of CH was performed by each investigator based on the criteria of the International Classification of Headache Disorder, 3rd Edition, beta version (ICHD-3β) [7]. Exclusion criteria were: inability to communicate in the Korean language, current enrollment in other clinical studies, and investigator’s judgment of cognitive or psychological difficulty to complete the questionnaire. In this study, homemakers, students, and patients without occupational experience were also excluded (Fig. 1). The study protocol was reviewed and approved by the local ethics committee or internal review board of each participating hospital, and all procedures were in line with the Declaration of Helsinki and Good Clinical Practice guidelines (2016–396-I). All patients were enrolled after informed written consent.

Two age and sex matched control groups were enrolled. All controls were aged between 19 and 65 years, with no history of diabetes, thyroid illness, severe obesity, severe hepatic or renal illness, malignancy, and they had the cognitive capability to complete the questionnaire. Patients with migraine or TTH were enrolled as headache controls. Healthy controls were recruited via notice board. Many were friends or relatives of patients with headaches or employees of the hospital. Additionally, healthy controls were required to be headache free (< 1 headache day per month) with no previous history of primary or secondary headache disorder based on the ICHD-3β [7]. All participants were enrolled after informed written consent.

### Clinical information and cluster headache questionnaire

Demographic features included age, sex, and lifestyle factors. Lifestyle factors, such as smoking and alcohol use, were assessed in all participants.

Investigators assessed and recorded clinical information regarding the current incidence and previous history of CH in the patients. Clinical information on current headaches included the location, severity, duration, and frequency of pain; associated symptoms, and duration of headache bouts. Previous history of CH was included, such as the duration from first CH bout, frequency of cluster periods, and pattern of recurrence.

### Occupation questionnaire and other parameters

All participants completed a questionnaire regarding their current employment status, shift-working time, weekly working hours, type of occupation, difficulties in working life, and satisfaction with occupation. To assess the impact of CH on occupation, any difficulties at work due to CH were assessed, such as failure to obtain or retain jobs, job changes (department or occupation), promotional disadvantage, voluntary resignation, reduction in productivity, low participation in out-of-work activities, and sick leave. We compared difficulties at work due to headaches between patients with CH and migraine or TTH, and difficulties at work were generally assessed in the headache-free controls.

Each patient completed a self-administered questionnaire assessing depression with the Patient Health Questionnaire-9 (PHQ-9), anxiety with the Generalized Anxiety Disorder-7 (GAD-7), and stress with the Short Form Perceived Stress Scale-4 (PSS 4) [8–11].

Each item on the PHQ-9 and GAD-7 was rated using a four-point scale (0 = never, 1 = several days, 2 = more than half the time, and 3 = nearly every day). Items were rated based on occurrence over the previous 2 weeks. The total PHQ-9 and GAD-7 scores ranged from 0 to 27 and 0 to 21, respectively [8, 9].

The PSS-4 consists of four items where respondents are required to rate how often they experienced stressful situations in the previous month on a Likert scale ranging from 0 to 4 (0 = never to 4 = very often). Two of the PSS-4 items were recorded due to the reversed scale. Higher scores denoted higher stress levels [10].

### Statistics

Chi-square and Student *t*-tests were used to compare nominal and continuous variables, respectively. Kolmogorov–Smirnov tests were performed to determine the normality of variable distribution. When normality was not confirmed, continuous variables were analyzed using the Mann-Whitney or Kruskal Wallis tests. *p* < 0.05 was considered statistically significant. Logistic regression was performed adjusting for age, sex, and PHQ-9, GAD-7, and PSS-4 scores as predictors for any difficulty at work or sick leave. Data were processed using IBM SPSS Statistics software (version 20.0 for Windows, IBM Corp., Armonk, NY, USA).

## Results

We initially enrolled 159 patients with CH, 40 patients with migraine or TTH, and 53 headache-free controls. Of the 159 patients with CH, 142 (91.0%) were surveyed during the cluster period. Participants with incomplete questionnaires or without occupational experience were excluded (Fig. 1). Following this, the questionnaires of 143 patients with CH (CH, *n* = 19, episodic CH, *n* = 100; chronic CH, *n* = 5; probable CH, *n* = 19), 38 patients with migraine or TTH (chronic migraine, *n* = 5; episodic migraine, *n* = 25; chronic TTH, *n* = 4; episodic TTH, *n* = 4), and 52 controls were analyzed.

### Entire study population analysis

The mean age of patients with CH was 38.1 ± 9.6 years, and 124 patients were male (86.7%); there were no differences in age and sex distribution among patients with CH, migraine or TTH, and headache-free controls (Table 1). The proportion of individuals who had retired was higher in the CH group than in the other groups (CH: 7.7%, Migraine/TTH: 5.3%, Control: 0%; *p* = 0.029). Among the 11 patients with CH who were retired, five had resigned from their job due to CH. Among the patients with CH, 25 were employers or self-employed, 96 were employees, and 22 were freelancers. The proportion of employees was lower in the CH group than in the other groups (CH: 67.6%, Migraine/TTH: 84.2%, Control: 96.2%; *p* = 0.001). There were no significant differences in shift working time, weekly working hours, and job satisfaction among the three groups (Table 1). Variable job burdens due to CH were reported in the CH group: two patients reported failure to obtain a job (1.4%), seven patients changed department or occupation (4.9%), and 17 patients had been dismissed or had voluntarily resigned (11.9%).

**Table 1:** Demographic characteristics, employment patterns, and satisfaction with occupation in patients with cluster headache, patients with migraine or TTH, and controls

	Cluster headache (*n* = 143)	Migraine or TTH (*n* = 38)	Controls (*n* = 52)	*P*-value
Male, n (%)	124 (86.7)	32 (84.2)	43 (82.7)	0.761
Age (year)	38.1 ± 9.6	37.6 ± 10.2	35.3 ± 8.9	0.193
Retirement, n, (%)	11 (7.7)	2 (5.3)	0 (0)	0.029
Employment status, n (%)				0.001
Employer/self-employment	25 (17.6)	3 (7.9)	1 (1.9)	
Employee	96 (67.6)	32 (84.2)	50 (96.2)	
Freelancer/others	22 (15.4)	3 (7.9)	1 (1.9)	
Shift working time, n (%)				0.781
Daytime only	96 (67.6)	25 (65.8)	34 (65.4)	
Nighttime only	5 (3.5)	3 (7.9)	2 (3.8)	
Day & Night time	23 (16.3)	5 (13.2)	6 (11.5)	
Etc	18 (12.7)	5 (13.2)	10 (19.2)	
Working hours/ week				0.151*
40 h	27 (19.6)	6 (15.8)	8 (15.4)	
40–52 h	66 (47.8)	14 (36.8)	33 (63.5)	
52–60 h	23 (16.7)	11 (28.9)	5 (9.6)	
> 60 h	22 (15.9)	7 (18.4)	6 (11.5)	
Job satisfaction, n (%)				0.295^†^
Satisfaction	77 (64.2)	16 (51.6)	33 (64.7)	
Neutral	38 (31.7)	11 (35.5)	17 (33.3)	
Dissatisfaction	5 (4.2)	4 (12.9)	1 (2.0)	

*TTH* tension-type headache

^*^5 patients with cluster headache did not give any information about working hours; ^†^31 patients give no response

### Analysis of the 220 employed patients

Among the participants who were currently employed, patients with CH more frequently experienced difficulties at work (CH: 84.8%, Migraine/TTH: 63.9%, Control: 36.5%; *p* < 0.001), reductions in productivity (CH: 60.6%, Migraine/TTH: 33.3%, Control: 11.5%; *p* < 0.001), low participation (CH: 36.4%, Migraine/TTH: 13.9%, Control: 5.8%; *p* < 0.001), and required sick leave (CH: 39.4%, Migraine/TTH: 13.9%, Control: 3.8%; *p* < 0.001) than patients with migraine or TTH and headache-free controls (Table 2). Multiple logistic regression, adjusting for age, sex, and PHQ-9, GAD-7, and PSS-4 scores, was used to assess any predictors for difficulties at work and found that CH and migraine or TTH were associated with increased risk (odds ratios: CH: 8.262, Migraine/TTH: 3.05). Multivariable logistic regression, adjusting for age, sex, and PHQ-9, GAD-7, and PSS-4 scores, was used to assess any predictors of sick leave and found that CH was associated with increased risk (odds ratio 15.12, Table 3).

**Table 2:** Job burden, depression, anxiety, and stress profiles in patients with cluster headache, patients with migraine or TTH, and controls among patients with current job

	Cluster headache (*n* = 132)	Migraine or TTH (*n* = 36)	Controls^a^ (*n* = 52)	*P*-value
Any difficulty at work	112 (84.8)	23 (63.9)	19 (36.5)	< 0.001
Fail to get or lose job	4 (3)	1 (2.8)	7 (13.5)	0.029
Changed job	3 (2.3)	0	4 (7.7)	0.083
Promotion disadvantage	0	1 (2.8)	2 (3.8)	0.093
Voluntary resignation	10 (7.6)	0	6 (11.5)	0.036
Reduced ability	80 (60.6)	12 (33.3)	6 (11.5)	< 0.001
Low participation	48 (36.4)	5 (13.9)	3 (5.8)	< 0.001
Sick absence	52 (39.4)	5 (13.9)	2 (3.8)	< 0.001
PHQ-9	7.4 ± 6.4	6.2 ± 5.3	2.6 ± 2.5	< 0.001
GAD-7	7.3 ± 5.3	5.7 ± 4.3	1.9 ± 2.2	< 0.001
PSS-4	6.5 ± 2.9	6.4 ± 3.5	5.3 ± 2.2	0.022

*TTH* tension-type headache, *GAD-7* Generalized Anxiety Dirorder-7, *PHQ-9* Patient Health Questionnaire-9, *PSS-4* Perceived Stress Scale-4

^a^Any difficulty at work was generally assessed in the controls; Data was presented as n (%) or mean ± standard deviation

**Table 3:** Multivariable logistic analyses about predictors for difficulty at work and sick absence among 220 participants with current job

	Any difficulty at work		Sick leave	
	OR (95% CI)	*p*-value	OR (95% CI)	*p*-value
Age	0.94 (0.90–0.98)	0.002	0.96 (0.92–1.00)	0.041
Sex, women	0.29 (0.11–0.74)	0.010	1.80 (0.70–4.63)	0.220
Groups				
Controls	1		1	
Migraine or TTH	3.05 (1.10–8.49)	0.032	3.85 (0.67–21.98)	0.130
Cluster Headache	8.26 (3.36–20.30)	< 0.001	15.12 (3.28–69.74)	< 0.001

Adjusted for depression by Patient Health Questionnaire-9, anxiety by Generalized Anxiety Dirorder-7, stress by Perceived Stress Scale-4

*TTH* tension-type headache

### Clinical features of patients with CH and sick leave analysis

Among the 132 patients with CH (CH, *n* = 17; episodic CH, *n* = 93; chronic CH, *n* = 5; probable CH, *n* = 17), the patients who required sick leave were younger at CH onset than those who did not (25.8 years vs. 28.7 years, *p* = 0.014). Pain severity, as measured by the visual analogue scale (VAS) was 9.3 ± 1.3 in patients with CH that required sick leave and 8.8 ± 1.2 in those who did not. Comparing sick leave with diurnal rhythms, the patients with CH with diurnal periodicity during the daytime more frequently required sick leave than those without periodicity or with periodicity during the night time (Table 4, *p* = 0.003). There was no significant difference in sick leave associated with sex, age, CH subtypes, CH recurrence, or type of employment (Table 4).

**Table 4:** Difference of cluster features according to experience of sick leave among 132 CH patients with current job

	Total (*n* = 132)	Sick leave		*p*-value
		Present (*n* = 52)	Absent (*n* = 80)	
Male	115 (87.1)	42 (80.8)	73 (91.3)	0.079
Age (year)	37.2 ± 8.7	35.9 ± 8.5	38.0 ± 8.8	0.179
Onset age of CH	28.7 ± 11.2	25.8 ± 11.3	30.6 ± 10.8	0.014
Duration of cluster headache, year	7.8 ± 11.6	10.2 ± 8.5	7.4 ± 7.5	0.057
Recurrence	103 (78)	43 (82.7)	60 (75.0)	0.297
Cluster bout	117 (88.6)	48 (92.3)	69 (86.3)	0.402
Frequency of CH/day	2.1 ± 1.8	2.0 ± 1.7	2.1 ± 1.9	0.629
Duration of CH, min	104.5 ± 73.4	115.7 ± 91.0	91.0 ± 12.6	0.271
Pain severity, VAS	9.0 ± 1.2	9.3 ± 1.3	8.8 ± 1.2	0.008
Chronic CH	5 (3.8)	3 (5.8)	2 (2.5)	0.382
Probable CH	17 (12.9)	4 (7.7)	13 (16.3)	0.189
Total bouts	8.0 ± 11.6	8.2 ± 8.3	7.6 ± 7.5	0.783
Diurnal periodicity^a^				0.003
None	64 (49.6)	17 (33.3)	47 (60.3)	
Day (6:00–17:59)	33 (25.6)	20 (39.2)	13 (16.7)	
Night (18:00–05:59)	27 (20.9)	10 (19.6)	17 (21.8)	
Both time	5 (3.9)	4 (7.8)	1 (1.3)	
Employment status				0.436
Employer/self-employment	22 (16.7)	6 (11.5)	16 (20)	
Employee	90 (68.2)	38 (73.1)	52 (65)	
Freelancer/others	20 (15.2)	8 (15.4)	12 (15)	

Data was presented as n (%) or mean ± standard deviation

*CH* cluster headache, *VAS* visual analogue scale

^a^3 patients not give any information

Multivariable logistic regression showed that severe pain (VAS ≥ 9) and diurnal periodicity during the daytime were significant predictors of sick leave after adjusting for age, onset age of CH, sex, PHQ-9, GAD-7, and PSS-4 scores; and cluster year (Additional file 1: Table S1).

## Discussion

The purpose of this study was to investigate the employment status and job burden of patients with CH. The main findings of this study were follows: 1) more patients were self-employed and less were employees in the CH group than in the other groups; 2) patients with CH had a 8.26× increased risk of having difficulties at work and a 15.12× increased risk of requiring sick leave compared with headache-free controls after adjusting for age, sex, and depression, anxiety, and stress levels; 3) and, in the CH group, the patients requiring sick leave were younger at CH onset and had more severe pain than those who did not require sick leave.

We found that patients with CH are more frequently self-employed than controls. This is consistent with the previous studies about the condition of CH [12–14]. One study reported that a greater proportion of patients with CH work full-time compared to controls without headaches; however, there was a high ratio of male patients with CH in that study, which may have biased the results [15]. There was no difference in working time, weekly working hours, or job satisfaction between the CH group and age-sex matched controls. The clustering of severe and painful attacks may influence patterns of employment and require greater personal responsibility; however, the reasons behind or the consequences of this pattern could not be clarified with this cross-sectional study setting.

In this study, 84.8% of patients with CH complained of difficulties in working life and over one third reported reductions in productivity and low participation. The rate of sick leave requirement in patients with CH was reported at 29.6% in Denmark, 68% in the US, and 39.4% in this study [3, 4]. The cultural environment and socioeconomic status may influence the sick leave rate, and 39.4% of patients with CH had 10× higher sick leave rates than controls. Solomon et al. found that patients with CH had significantly lower rates of social activity compared with patients with migraines, in line with our findings [16]. Indirect costs from work disability and lower participation caused by CH impose a significant socioeconomic burden on patients and society [17, 18].

Psychiatric comorbidities, such as anxiety, depression, panic attacks, or suicidal ideation are prevalent and severe in patients with CH, especially during cluster periods or chronic CH [19, 20]. Similar to the findings of previous studies, patients with CH in our study complained of higher levels of anxiety, depression, and stress when compared with patients with migraine or TTH and controls. We found higher PHQ-9, GAD-7, and PSS-4 scores in patients with CH; however, after adjustment for these confounding variables, CH still increased the risk of having difficulties at work and sick leave. This result was similar to that of a previous study that found an association between severe and persistent migraines and increased risk of work disabilities, after adjusting for mental disorders [21].

This is the first study to analyze predictors of sick leave among patients with CH. Our subgroup analysis of CH showed that sick leave was significantly associated with a younger age at CH onset, pain severity, diurnal periodicity during the daytime. Although the significance of younger age at CH onset was decreased with multivariable logistic analysis, the association between younger age at onset and increased risk for sick leave suggested that the headaches in this subgroup started before they had the opportunity to secure meaningful employment and thus these patients were more disabled by their condition when they started working and/or less able to adapt to the working environment.

This study has several limitations. First, the degree of occupational satisfaction, sick leave, and reductions in productivity were not examined using a scale. These data were collected by questionnaire; therefore, it was difficult to estimate the amount of sick leave or disability at the time the headaches were experienced based on participant recollection. Second, the headache control group included the migraine and TTH groups, and job burden may have varied among headache controls. The sex ratio among migraineurs in this study differed from that among the actual patient population, and thus our sample of patients with migraine may not have been representative. Third, headache-free controls were recruited among the relatives of patients, volunteers, and hospital staff and their families and required stricter exclusion criteria (no history of diabetes, thyroid illness, severe obesity, severe hepatic or renal illness, malignancy, and cognitive capability to complete the questionnaire). This may have led to sampling bias, which could have influenced the number of retirees in the control group. Additionally, the job burden analysis was only conducted using data from participants with current jobs. There was a disadvantage that job burden due to headache among patients who were employed was compared to overall job burden among the healthy controls. This study was conducted based on the ICHD-3β criteria and could not include a sufficient number of patients with chronic CH to analyze the impact of chronic CH. Similar to this study, the frequency of chronic CH has been reported at 3.5% in Japan [22]. These data reflect a substantial job burden for Asian patients with CH, despite the low proportion of patients with chronic CH.

## Conclusions

This study reported the effect of CH on occupational factors and compared age-sex matched patients with patients with other types of headache and headache-free controls. In addition, we revealed that CH were an important predictor of work disability and need for sick leave after adjusting for psychiatric comorbidities. Furthermore, we revealed that severity of pain, younger age at CH onset, and diurnal periodicity during the daytime were associated with sick leave of CH patients.

## Additional file

Additional file 1: **Table S1**. Multivariable logistic analysis for sick leave in CH patients with current job. (DOCX 17 kb)

Additional file 2: Data file of 233 participants with 133 variables. (SAV 308 kb)

## Abbreviations

CH: Cluster headache
GAD-7: Anxiety by the generalized anxiety dirorder-7
ICHD-3β: International classification of headache disorder, 3rd edition, beta version
PHQ-9: Patient health quetionnare-9
PSS 4: Stress by short form perceived stress scale-4
TTH: Tension-type headache
