# Supplementary material for: Impact of cluster headache on employment status and job burden: a prospective cross-sectional multicenter study
Source: J Headache Pain. 2018 Sep 3;19(1):78. doi: 10.1186/s10194-018-0911-x (PMC6120854; doi:10.1186/s10194-018-0911-x)
Supplement: Supplementary file 1 — Table S1. Multivariable logistic analysis for sick leave in CH patients with current job. (DOCX 17 kb) [file 10194_2018_911_MOESM1_ESM.docx]

|  | Model 1 | | Model 2* | |
| --- | --- | --- | --- | --- |
|  | P-value | OR (95% CI) | P-value | OR (95% CI) |
| Younger onset age of CH (< 20 years old) | 0.050 | 2.53 (1.00-6.39) | 0.148 | 2.67 (0.71-11.00) |
| Severe pain (≥VAS 9) | 0.004 | 3.96 (1.57-10.00) | 0.005 | 4.29 (1.57-11.77) |
| Diurnal periodicity^+^ | 0.002 |  | 0.002 |  |
| None or night |  | reference |  | reference |
| Day or both |  | 3.99 (1.68-9.48) |  | 4.17 (1.67-10.40) |

Additional file 1: Table S1. Multivariable logistic analysis for sick leave in CH patients with current job

CH, cluster headache; VAS, visual analogue scale; Model 2 was adjusted age, sex, depression by Patient Health Questionnaire-9, anxiety by Generalized Anxiety Dirorder-7, stress by Perceived Stress Scale-4 and cluster year.
